# Physical Activity, Screen Time, and Emotional Well-Being during the 2019 Novel Coronavirus Outbreak in China

**DOI:** 10.3390/ijerph17145170

**Published:** 2020-07-17

**Authors:** Fei Qin, Yiqing Song, George P Nassis, Lina Zhao, Yanan Dong, Cuicui Zhao, Yiwei Feng, Jiexiu Zhao

**Affiliations:** 1 School of Physical Education, Jinan University, Guangzhou 510632, China; qinfei@ciss.cn; 2 Exercise Biology Center, China Institute of Sport Science, Beijing 100061, China; gnassis@health.sdu.dk (G.P.N.); eve314@163.com (L.Z.); 17865515266@163.com (C.Z.); 15888284143@163.com (Y.F.); 3 Department of Epidemiology, Indiana University Richard M. Fairbanks School of Public Health, Indianapolis, IN 46202, USA; yiqsong@iu.edu; 4 Department of Sports Science and Clinical Biomechanics, Faculty of Health Sciences, University of Southern Denmark, 5230 Odense, Denmark; 5 School of Physical Education and Sport Training, Shanghai University of Sports, Shanghai 200438, China; 6 School of Kinesiology, Shanghai University of Sports, Shanghai 200438, China; 7 Beijing Institute of Sports Science, Beijing 100075, China; 15092692183@163.com; 8 Department of Physical Education, Qufu Normal University, Qufu 273165, China

**Keywords:** COVID-19, lockdown, sedentary lifestyle, physical activity, screen time, psychological impact

## Abstract

We aimed to evaluate the effects of the COVID-19 lock down on lifestyle in China during the initial stage of the pandemic. A questionnaire was distributed to Chinese adults living in 31 provinces of China via the internet using a snowball sampling strategy. Information on 7-day physical activity recall, screen time, and emotional state were collected between January 24 and February 2, 2020. ANOVA, χ² test, and Spearman’s correlation coefficients were used for statistical analysis. 12,107 participants aged 18–80 years were included. During the initial phase of the COVID-19 outbreak, nearly 60% of Chinese adults had inadequate physical activity (95% CI 56.6%–58.3%), which was more than twice the global prevalence (27.5%, 25.0%–32.2%). Their mean screen time was more than 4 hours per day while staying at home (261.3 ± 189.8 min per day), and the longest screen time was found in young adults (305.6 ± 217.5 min per day). We found a positive and significant correlation between provincial proportions of confirmed COVID-19 cases and negative affect scores (r = 0.501, *p* = 0.004). Individuals with vigorous physical activity appeared to have a better emotional state and less screen time than those with light physical activity. During this nationwide lockdown, more than half of Chinese adults temporarily adopted a sedentary lifestyle with insufficient physical activity, more screen time, and poor emotional state, which may carry considerable health risks. Promotion of home-based self-exercise can potentially help improve health and wellness.

## 1. Introduction

In late 2019, new cases of Corona Virus Disease-19 (COVID-19) were reported in Wuhan, Hubei province, China [1]. COVID-19, like SARS, it is caused by a beta-coronavirus that can spread though human-to-human transmission [2]. There is currently no effective vaccine for the prevention of COVID-19. As of 8 June 2020, more than 6,931,000 cases have been reported to the World Health Organization (WHO), from 216 countries and territories [3]. 

In response, beginning from 23 January 2020, the Chinese government initiated a series of prevention and control strategies to curb the spread of the virus, such as locking down entire cities, and travel warnings, home-based medical observation, and home quarantine [4,5]. Furthermore, national holidays were extended and family reunions discouraged [6]. Unavoidably, restrictions on travel and outdoor activities disrupted the lifestyle and routine daily activities of Chinese residents. Thus, people were more likely to experience insufficient physical activity, increased screen time, and greater anxiety, which may carry considerable risks to health and well-being [7,8].

It is well known that insufficient physical activity is a key risk factor for cardiovascular disease, hypertension, diabetes, and prostate and colon cancer [9,10]. Since the beginning of the COVID-19 outbreak, some scholars have proposed that there is a strong rationale behind continuing physical activity in the home: to stay healthy and maintain immune system function in the current precarious environment [7]. Therefore, a series of crucial questions need to be answered: Is individual physical activity affected during periods of epidemic prevention and control? What is the prevalence of insufficient physical activity among Chinese residents in different areas?

Research published since the COVID-19 outbreak has focused on epidemiological prediction [11], clinical characteristics of patients [2,12], the genomic sequence of the virus [13], and investigations of the psychological state of the population at large [8]. However, because the COVID-19 crisis confined us to our homes, information on lifestyle among the general population in China has been scarce. The aim of this study was to investigate levels and characteristics of lifestyle among Chinese residents during the home quarantine induced by COVID-19. Investigations like this one are essential to provide evidence to inform policy makers and guide future policy and programmed planning for health promotion, given the institution of home-based medical observation.

## 2. Materials and Methods

### 2.1. Study Population

This is a nationwide cross-sectional study, which aimed to survey changes in activity and daily routine among Chinese citizens during home quarantine. We recruited Chinese adults living in 31 provinces of China during Chinese New Year public holiday (24 January through 2 February 2020), which coincided with the initial stages and outbreak peak of the COVID-19 epidemic. The questionnaire was distributed using a snowball sampling strategy through contactless distribution on the internet. Individuals were asked to recall their physical activity and emotional state over the past 7 days, within an overall collection period between 31 January and 9 February 2020. Instructors in social sports in various provinces participated in questionnaire distribution via their working platforms. In addition, the WeChat Subscription and social media platforms with high click-through rates also reprinted our questionnaire. The sample size of each province with quotas based on the sampling plan of the sixth national physical fitness monitoring, and the sample size of all provinces, was divided into four levels according to population density (Table A1). Data analyses excluded Taiwan, Hong Kong, and Macau because they are not covered by the physical fitness monitoring points system. Full ethical approval was obtained from the China Institute of Sport Science, Beijing, China (CISS-2020-01-28). All participants gave informed consent.

### 2.2. Procedures

Physical activity data and sedentary time were collected using the Chinese validated version of the International Physical Activity Questionnaire (IPAQ)-Short form [14], and translated from the Chinese version into Tibetan (termed double translation/back translation), for use in Tibet and Qinghai. The form asks participants to recall, over the last seven days, the number of days per week and minutes per day spent engaging in vigorous physical activity, moderate physical activity, and walking. Similarly, the time spent sitting over the past seven days was also asked. Daily total physical activity (MET-min-day) was calculated. Meanwhile, based on the guidelines for data processing and analyses of the IPAQ [15] and the WHO recommended guidelines [16], three different levels (vigorous, moderate, and light) of activity were reported (Table A2). The prevalence of insufficient physical activity, according to current WHO guidelines, which recommended that adults engage in at least 75 minutes of vigorous-intensity physical activity per week, or at least 150 min of moderate physical activity, or any equivalent combination of the two [16]. All data were managed and logic-screened by standard methods according to the guidelines for data processing and analyses of the IPAQ [15]. 

Mood was assessed using the Positive and Negative Affect Schedule (PANAS) questionnaire of two 10-item scales [17]. Ten items measured positive affect (e.g., excited, inspired) and 10 items measured negative affect (e.g., upset, afraid). Each item was rated on a five-point Likert Scale, ranging from 1 (very slightly or not at all) to 5 (extremely), to assess the extent to which the participant felt the listed positive or negative emotion over past week. Scores for the positive and negative affect scales were summed (range10–50), with higher scores on both subscales representing greater positive or negative mood, respectively. 

We considered four relevant sociodemographic factors in our analysis: place (urban and rural regions), sex (men and women), age group, and geographical location (province). Age was divided into ten groups: <20 years, 20–24 years, 25–29 years, 30–34 years, 35–39 years, 40–44 years, 45–49 years, 50–54 years, 55–59 years, and ≥60 years. Furthermore, in order to clarify the correlations of physical activity, screen time, and mood of individuals during the epidemic, the three physical activity levels were analyzed with screen time and mood state. 

### 2.3. Statistical Analysis

Descriptive statistics such as percentages, 95% CIs, means, and standard deviations, were calculated for categorical variables and continuous variables, respectively. The χ² test was used to assess statistical difference in the prevalence of insufficient physical activity according to region of residence (urban and rural regions), sex (men and women), and age subgroups. The normality of continuous variables was checked by the Kolmogorov–Smirnov test. The differences in screen time, positive affect scores, and negative affect scores among region of residence (urban and rural regions), sex (men and women), age subgroups, and three intensity levels of physical activity were tested using ANOVA. The level of *p* < 0.05 was defined as statistical significance. Our overall prevalence estimates were compared with the global age-standardized prevalence of insufficient physical activity and China’s prevalence of insufficient physical activity (non-epidemic period) from WHO [18]. Given the available sample size information only for the total global population [18], we were able to perform a χ² test to determine the statistical significance of the difference between the prevalence of insufficient physical activity in China and the global levels. Spearman’s correlation coefficients were calculated to assess the relationship of provincial proportions of confirmed COVID-19 cases with the prevalence of insufficient physical activity and the means of screen time and PANAS scores, separately, in 31 provinces in mainland China. The proportion of COVID-19 cases was calculated by dividing the total number of confirmed COVID-19 cases (as of 3 February 2020) by the number of the total population (as of the end of 2018) for each of the 31 provinces. Provincial populations at the end of 2018 are cited from the China Statistical Yearbook published by the National Bureau of Statistics of China (2019). All data were analyzed with SPSS software 22.0 (IBM Inst., Chicago, IL, USA). 

## 3. Results

### 3.1. Survey Respondents

**Figure 1:**
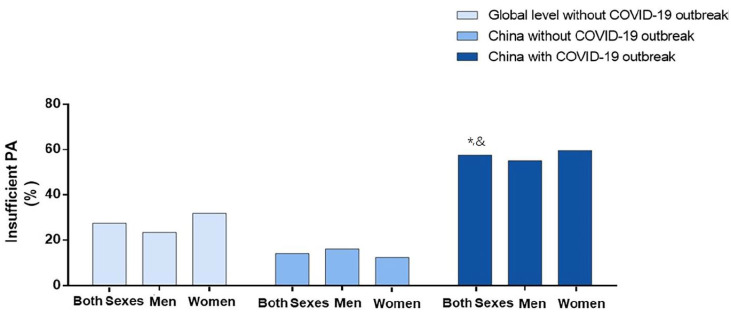
Prevalence of insufficient physical activity (PA) among Chinese adults aged 18 and over during COVID-19 epidemic period in China, compared with the national levels in China (during non-epidemic period) and the average global level (World Health Organization (WHO) data, without COVID-19 outbreak), among all participants, men, and women, separately. PA = physical activity. * *p* < 0.05 versus Global level; ^&^ *p* < 0.05 versus China without COVID-19 outbreak (non-epidemic period).

During the home quarantine required by the COVID-19 outbreak, 12,107 participants (5633 men and 6474 women) were included in the final analysis (Table 1). Across all regions of China, 57.5% (95% CI 56.6%–58.3%) of individuals were insufficiently physically active during home quarantine (Figure 1). 

### 3.2. Physical Activity Impact

Compared with the global level reported by WHO, the prevalence of insufficient physical activity more than doubled during the initial stage of COVID-19 epidemic in China (global: 27.5% vs. China in epidemic stage: 57.5%, χ^2^ = 13,088.10, *p* < 0.0001; Figure 1). Meanwhile, compared with the physical activity of Chinese residents during a non-epidemic period reported by WHO (14.1%), the prevalence of insufficient physical activity rose over 3-fold in China during the epidemic quarantine (57.5%, χ^2^ = 12,700.00, *p* < 0.0001; Figure 1). In addition, compared with global and Chinese averages (non-epidemic period), the percentage of men engaging in insufficient physical activity was 31.7% and 39.1% higher, respectively, during the initial stage of epidemic in China (Figure 1). Meanwhile, compared with global and Chinese averages (non-epidemic period), the percentage of women engaging in insufficient physical activity was 27.8% and 47.3% higher, respectively, during the initial stage of the epidemic in China (Figure 1).

**Figure 2:**
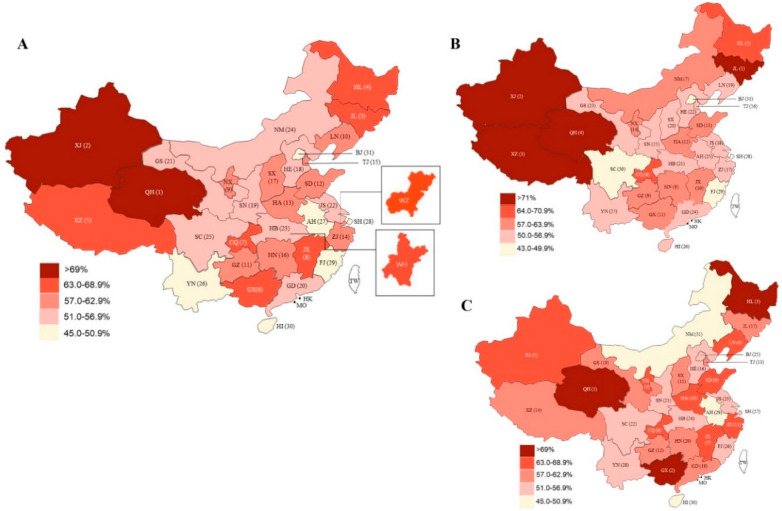
Provincial proportions of insufficient physical activity during home quarantine induced by COVID-19 for 31 provinces in mainland China. A: both sexes; B: men; C: women. BJ = Beijing. TJ = Tianjin. HE = Hebei. SX = Shanxi. NM = Inner Mongolia. LN = Liaoning. JL = Jilin. HL = Heilongjiang. SH = Shanghai. JS = Jiangsu. ZJ = Zhejiang. AH = Anhui. FJ = Fujian. JX = Jiangxi. SD = Shandong. HA = Henan. HB = Hubei. HN = Hunan. GD = Guangdong. GX = Guangxi. HI = Hainan. CQ = Chongqing. SC = Sichuan. GZ = Guizhou. YN = Yunnan. XZ = Tibet. SN = Shaanxi. GS = Gansu. QH = Qinghai. NX = Ningxia. XJ = Xinjiang. TW = Taiwan. HK = Hong Kong. MO = Macao. WH = Wuhan. WZ = Wenzhou.

The provincial prevalence of insufficient physical activity ranged from 48.9% in Beijing to 73.3% in Qinghai, the top five provinces being Qinghai, Xinjiang, Jilin, Heilongjiang, and Tibet. (Figure 2 and Table A3). In addition, focusing on two cities with serious epidemic situations, the prevalence of insufficient physical activity was 63.5% (53.8–73.1) in Wuhan, and 63.8% (58.2–69.1) in Wenzhou (Figure 2). Among men, four provinces had prevalence of insufficient activity over 71.0% (Jilin, Xinjiang, Tibet, and Qinghai) (Figure 2 and Table A3). Among women, the prevalence of insufficient physical activity was the highest in Qinghai, followed by Guangxi (Figure 2 and Table A3). 

By gender, the prevalence of insufficient physical activity among men (55.1%, 53.8–56.4) was significantly lower than among women (59.5%, 58.2–60.7) during home quarantine (χ^2^ = 29.51, *p* < 0.0001; Table 2). In addition, the rate of participation in vigorous activity was higher among men than among women (χ^2^ = 29.51, *p* < 0.0001; Table 2). Across age groups, the group with the highest prevalence of insufficient physical activity was young adults aged 20–34 years (χ^2^ = 275.87, *p* < 0.0001; Table 2). In contrast, a lower prevalence of insufficient physical activity was found in those aged 55–59 years (41.1%, 35.4–46.8) and over 60 years old (41.3%, 34.2–48.9) during home quarantine induced by COVID-19 (Table 2).

### 3.3. Screen Time Impact

**Figure 3:**
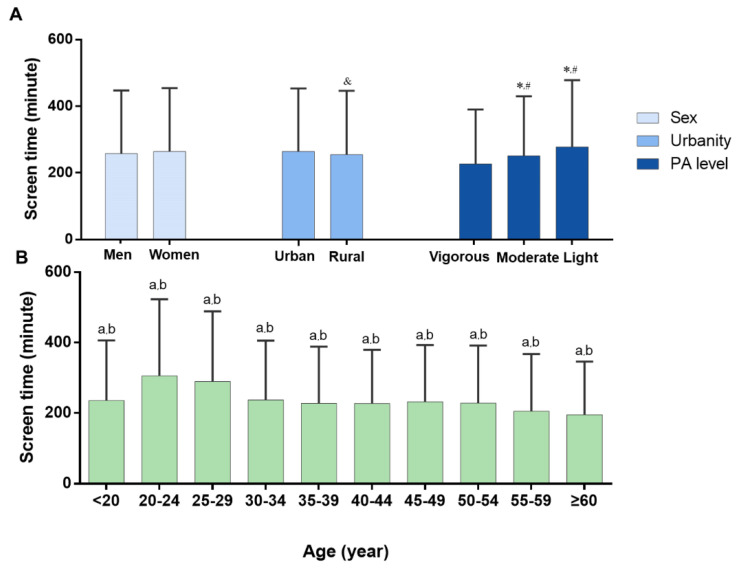
Screen time among Chinese residents aged 18 years and over during home quarantine induced by COVID-19, 2020. (**A**): Comparisons in screen time by sex, urban or rural residence, and physical activity level. (**B**): Comparisons in screen time by age. PA = physical activity. ^&^ *p* < 0.05 versus Urban; * *p* < 0.05 versus Vigorous level; ^#^ *p* < 0.05 versus Moderate level; ^a^ *p* < 0.05 versus 20–24; ^b^ *p* < 0.05 versus 25–29. All values were presented as mean ± SD.

Screen time among Chinese residents was 261.3 ± 189.8 min during home quarantine. Across age groups, the longest screen times were in young adults aged 20–24 (305.6 ± 217.5 min, *p* < 0.0001) and 25–29 group (289.9 ± 198.9, *p* < 0.0001; Figure 3B). Not surprisingly, screen time increased as physical activity level declined (vigorous vs moderate vs light: 226.7 ± 163.4 min vs. 251.4 ± 178.4 min vs. 277.7 ± 200.7 min, *p* < 0.0001, Figure 3A). 

### 3.4. Emotional Well-Being Impact

Mean positive affect scores were higher among men than among women (25.09 ± 7.06 vs. 24.51 ± 6.70, *p* < 0.0001), while negative affect scores of men were somewhat lower (19.04 ± 7.00 vs. 19.61 ± 7.08, *p* < 0.0001, Table 3). In addition, the positive affect scores associated with vigorous physical activity were markedly higher than those associated with moderate and light levels (*p* < 0.0001), while there was a downward trend of negative affect scores from vigorous physical activity to light physical activity (*p* < 0.0001, Table 3).

### 3.5. Correlations of Provincial Levels of Lifestyle and Emotional State with Proportion of Confirmed COVID-19 Cases in 31 Provinces of Mainland China

**Figure 4:**
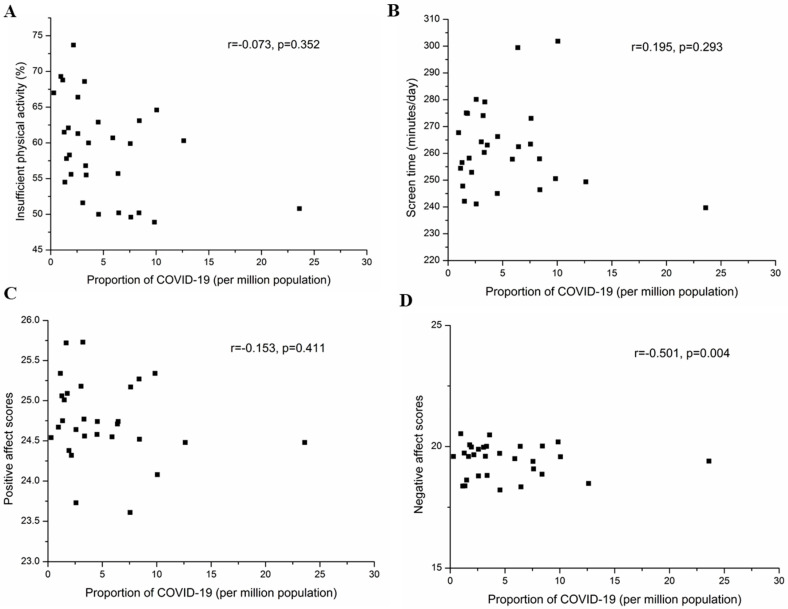
Scatterplots showing correlations between provincial levels of lifestyle and emotional state and provincial proportions of confirmed COVID-19 cases in 31 provinces of mainland China, 2020. (**A**): insufficient physical activity; (**B**): screen time; (**C**): positive affect scores; (**D**): negative affect scores. Proportion of COVID-19 cases was calculated by dividing the total number of confirmed COVID-19 cases (until 3 February 2020) by the number of total population (by the end of 2018) in each of 31 provinces. Populations at the end of 2018 in different provinces are cited from the China Statistical Yearbook published by the National Bureau of Statistics of China (2019).

In addition, we found a positive correlation between the provincial proportions of confirmed COVID-19 cases and negative affect scores (r = 0.501, *p* = 0.004, Figure 4D) but no significant correlations with provincial levels of insufficient physical activity (r = −0.073, *p* = 0.352, Figure 4A), the provincial means of screen time (r = 0.195, *p* = 0.293, Figure 4B), or positive affect scores (r = −0.153, *p* = 0.411, Figure 4C).

## 4. Discussion

Our nationwide survey data yielded three main findings. Firstly, nearly 60% of Chinese citizens engaged in inadequate physical activity, more than twice the global prevalence. Secondly, screen time exceeded 4 h per day during home stay. Thirdly, the proportion of confirmed COVID-19 cases significantly correlated with provincial negative affect scores. Individuals engaging in vigorous physical activity had better emotional states whereas the opposite was true for light physical activity. Our findings indicate the importance of attention to and promotion of home-based exercise and early exercise guidance for health promotion during such lockdowns. 

We collected the data between 24 January and 2 February 2020 for the following reasons: First, this period was the initial stage of the COVID-19 outbreak, by which home-based exercise and fitness suggestions/recommendations had not yet been promoted to the public. Therefore, we were able to truly estimate the impact of the public health emergency on physical activity. Second, the period between 24 January and 2 February 2020 is the Chinese New Year national holiday, whose main activities include family reunions, traveling, shopping, and visiting relatives and friends. However, those holiday arrangements were disrupted by the epidemic, with restrictions on travel and gatherings. Consequently, we considered that this an optimal period to observe the lifestyle changes related to the COVID-19 outbreak.

Overall, sedentary behavior and insufficient physical activity posed the main health risks during the initial stage of the COVID-19 outbreak in China. Recent studies have shown that prolonged periods of sitting were associated with reduced lipoprotein lipase activity, glucose tolerance, and decreased glucose-stimulated insulin secretion [10,19]. Moreover, low physical activity levels are associated with increased prevalence of anxiety [20]. Therefore, it is critically important to promote well-being by offering more suggestions and professional guidance for exercise and fitness to people staying at home through government health management platforms or mainstream media. 

Our study showed lower activity among women than among men during the initial stage of the COVID-19 outbreak. However, evidence supporting the opposite trend in China during the non-epidemic period has been reported by WHO. The reasons for the sex difference in activity are that men and women are on equal terms in the Chinese workplace, but women engaged in more household activity than men during non-epidemic periods. With the COVID-19 outbreak, men stayed at home and shared housework. In addition, consistent with previous research results, women tended to engage in lower-intensity activity than men [18,21]. Among the different age groups, young adults aged 20–34 years had the highest prevalence of insufficient physical activity. Conversely, based on the high consciousness of health management behaviors among China’s elderly population, a relatively lower proportion of insufficient physical activity and less screen time were observed in aged 55–59 years and over 60 years old during home quarantine [22]. In other words, young people were at higher risk of engaging in sedentary and unhealthy lifestyles during this public health emergency, which suggests that using innovative social media (microblog, WeChat, and short video platform) may be optimal approaches to help promote healthier behaviors among young adults. 

Despite the trend of lower physical activity in all 31 regions of China, we also found a wide variation in provincial prevalence of insufficient physical activity: lower than 50% in a few provinces and more than 70% in others. The five provinces most seriously affected by the COVID-19 outbreak (i.e., proportions of confirmed cases) were Hubei (54.8%), Guangdong (55.7%), Henan (60.7%), Zhejiang (60.3%), and Hunan (59.9%). Three of those provinces fell below the average in prevalence of insufficient activity in China during the epidemic. For the Hubei province, the area of most serious viral spread, the prevalence of insufficient physical activity was not as high as we expected. However, focusing on Wuhan city, the epicenter of the outbreak, the prevalence of insufficient physical activity was nearly 9% higher compared with that in the Hubei province. Meanwhile, another city seriously affected by the epidemic, Wenzhou, had a similar trend in the prevalence of insufficient physical activity compared with that in Zhejiang province (an increase of 3.5%). Hence, with the growing coronavirus crisis, people in outbreak areas are likely to experience fear of becoming sick, feelings of helplessness, and panic [8]. It would be extremely difficult for them to maintain their daily exercise or physical activity routines. Overall, we believe it is indispensable to provide professional exercise and fitness health guidelines to maintain the best possible public health and immunity in epidemic areas [7]. Interestingly, our study found no correlation between provincial levels of insufficient physical activity and proportion of COVID-19 in the 31 provinces of mainland China. This may suggest that physical activity is affected by a variety of complex factors during public health emergency. This phenomenon is worthy of further investigation. 

The prevalence of insufficient physical activity in first-tier cities of China less affected by COVID-19 such as Beijing (48.9%) and Shanghai (50.2%) were obviously lower than the average level of China during initial stages of the outbreak, and Beijing is the least affected area. This phenomenon may be associated with high economic and educational levels in Beijing [23,24]. It is worth mentioning that the prevalence of insufficient physical activity was relatively low in several provinces with more tourism-based cities such as Hainan (49.6%) and Yunnan (50.8%). These areas usually have temperate climate and pleasant natural environments, low population density and community settings with city gardens and parks that may motivate people to be physically active outside [25,26]. Previous studies have suggested that green space could reduce the likelihood of being overweight by offering suitable spaces that encourage physical activity [27,28]. Nowadays green exercise has been increasingly recognized as a positive contribution to health and wellbeing [29,30,31]. Meanwhile, our findings may indicate that there may be a potential benefit of green space on physical activity behavior, especially for the lifestyle changes with public health emergency. Hence, it is necessary for designers or researchers to evaluate the green space interventions when maintaining safe distances during the pandemic.

Unexpectedly, several provinces that were less strongly affected by COVID-19, but had strict home quarantine and travel restrictions, showed a high prevalence of insufficient physical activity: Qinghai (73.7%), Xinjiang (69.3%), Jilin (68.8%), Heilongjiang (68.6%), and Tibet (67.0%). Because Jilin and Heilongjiang are located in the cold northeast regions of China, the closure of winter sports facilities because of the outbreak may explain the high prevalence of insufficient physical activity. Qinghai, Xinjiang, and Tibet are in western areas, with relatively underdeveloped economies and low health educational levels. Facing a public health emergency, residents in these provinces may place limited value on exercise, or their exercise habits may be easily changed. One similar study reported that compared to regular school days, children and adolescents in Tibet engaged in significantly less physical activity on the weekend [32]. Moreover, based on the anxiety and panic induced by COVID-19, religious activities such as chanting, praying, and meditating may also have occupied their time. Therefore, climate characteristics, geographical or cultural differences, socio-economical levels, and education might affect engagement in physical activity [18,23]. 

Screen time among adults is a significant factor in assessing health behaviors. Screen time among Chinese residents was more than 4 hours per day during home stay. Several epidemiological studies indicated that 2 h/day on screen-based entertainment was associated with a 48% increased risk of all-cause mortality while 4 h/day with an approximately 125% increase in risk of cardiovascular disease (CVD) events [33]. Screen time exceeding 2 h/day was associated with a higher risk of depression, especially in the female population [34]. Therefore, a sedentary lifestyle is associated with multiple adverse health outcomes including obesity, type 2 diabetes, CVD, and depression in adults [34,35,36]. Additionally, due to the video games and webcasts that attract a large number of young people, the longest screen times were found in those aged 20–24 and 25–29 years. A longitudinal follow-up investigation found that TV watching at age of 23 years was independently associated with composite factors of metabolic (including HDL and BMI) and inflammatory biomarkers (including CRP) [33]. Importantly, we also confirmed that individuals who took part in vigorous physical activity spent less screen time. The result was similar to that in previous studies [37]. This also implies that encouraging physical activity may be an effective way to decrease screen time, especially among young adults.

Finally, there was a positive correlation between the proportion of confirmed COVID-19 cases with provincial negative affect scores, with the highest negative affect scores reported in Hubei province. During the initial stage of the COVID-19 outbreak, the higher negative affect scores may be associated with the fear of falling sick [8,38] or dying, feelings of helplessness [39], and leisure constraints [40,41]. Furthermore, we found individuals engaging in vigorous physical activity had better emotional states, whereas those doing light physical activity showed the opposite trend. Such positive regulatory effects of physical activity on emotion have been documented in previous studies [42,43]. Our results remind us that physical activity is an effective means to maintain physical and mental health and immunity and may help reduce social economic burden or healthcare burden during quarantine. 

Our study has several limitations. First, because of the limited resources available and the rapid onset of the COVID-19 outbreak, the snowball sampling strategy was adopted. Although our respondents are not nationally representative samples of Chinese adults, we made efforts to utilize the social platforms used by instructors in social sports in various provinces to ensure the diversity and demographic representativeness of participants. Second, there seems to be an oversampling of younger participants (aged ≤ 30 years), especially students in our surveyed population, due to the relatively high participation in social networks, finally diminishing the study generalizability. Third, self-reported data on physical activity may not be as accurate as assessment using accelerometers, although we chose a WHO-approved physical activity questionnaire (IPAQ-S) with high reliability and validity for the investigation. Fourth, we only had available data of global age-standardized prevalence of insufficient physical activity and prevalence of insufficient physical activity in China from WHO to assess the differences between the epidemic period and a non-epidemic period. Lastly, it would be necessary to conduct a prospective study on the same group of participants after the epidemic period to capture trends in physical activity over time. Notwithstanding the aforementioned limitations, this study provides invaluable information on physical activity status across 31 provinces in China during the initial stage of the COVID-19 epidemic. Most significantly, our findings indicate the necessity of early health promotion and fitness guidance during home quarantine. This could guide future policy and programmed planning for health promotion during future health emergencies.

## 5. Conclusions

In summary, during the initial phase of the COVID-19 outbreak, nearly 60% of Chinese citizens engaged in inadequate physical activity, while the prevalence of insufficient physical activity was more than double the global level. Screen time among Chinese residents was more than 4 hours per day during the quarantine, and the longest screen times were found in young adults. Women, young adults, and the residents of remote regions had a higher prevalence of insufficient physical activity. During home quarantine, there was a positive correlation between the proportion of confirmed COVID-19 cases with provincial negative affect scores, and individuals engaging in vigorous physical activity had better emotional states and less screen time while the group engaging in only light activity showed the opposite trend. If the trends noted during the outbreak continue, the risk of chronic diseases in Chinese residents may be increased. 

## Figures and Tables

**Table 1:** Characteristics of the Chinese adults aged 18 and over during home quarantine induced by COVID-19 outbreak in China, 2020 (*n* = 12,107 participants aged 18–80 years old).

	Men	Women	Total
**Percentage *n* (%)**	5366 (46.5%)	6474 (53.5%)	12,107 (100%)
**Age (years)**			
<20	464 (8.2%)	390 (6.0%)	854 (7.1%)
20–24	1832 (32.5%)	2064 (31.9%)	3896 (32.2%)
25–29	644 (11.4%)	817 (12.6%)	1461 (12.1%)
30–34	568 (10.1%)	860 (13.3%)	1428 (11.8%)
35–39	608 (10.8%)	777 (12.0%)	1385 (11.4%)
40–44	570 (10.1%)	658 (10.2%)	1228 (10.1%)
45–49	463 (8.2%)	447 (6.9%)	910 (7.5%)
50–54	243 (4.3%)	238 (3.7%)	481 (4.0%)
55–59	148 (2.6%)	132 (2.0%)	280 (2.3%)
≥60	93 (1.7%)	91 (1.4%)	184 (1.5%)
**Urbanity**			
Urban regions	1751 (31.1%)	1949 (30.1%)	3700 (30.6%)
Rural regions	3882 (68.9%)	4525 (69.9%)	8407 (69.4%)
**Education**			
Primary school or lower	187 (3.3%)	161 (2.5%)	348 (2.9%)
Middle school	272 (4.8%)	413 (6.4%)	685 (5.7%)
High school	504 (8.9%)	700 (10.8%)	1204 (9.9%)
College	3260 (57.9%)	3707 (57.2%)	6963 (57.5%)
Graduate	1410 (25.0%)	1497 (23.1%)	2907 (24.0%)
Occupation			
Full-time student	2211 (39.3%)	2249 (34.7%)	4460 (36.8%)
Labor	458 (8.1%)	504 (7.8%)	962 (7.9%)
Professional	2280 (40.4%)	2476 (38.2%)	4756 (39.3%)
Unemployed and freelance	684 (12.1%)	1245 (19.2%)	1929 (15.9%)

**Table 2:** Intensity levels of physical activity stratified by sex, age, and urban or rural residence during home quarantine induced by COVID-19 outbreak in mainland China, 2020.

	Vigorous	Moderate	Light	*p* for Difference *
**Sex**				<0.0001
Men	23.0% (21.9–24.2)	21.9% (20.8–23.0)	55.1% (53.8–56.4)	
Women	19.4% (18.4–20.3)	21.2% (20.2–22.2)	59.5% (58.2–60.7)	
**Age**				<0.0001
<20	28.9% (25.8–32.0)	20.7% (18.0–23.5)	50.4% (47.0–53.7)	
20–24	17.1% (15.9–18.4)	18.7% (17.5–19.9)	64.2% (62.7–65.8)	
25–29	17.1% (15.0–19.1)	19.4% (17.4–21.5)	63.4% (61.0–65.9)	
30–34	17.9% (15.8–19.7)	22.1% (20.0–24.4)	60.0% (57.6–62.5)	
35–39	23.3% (21.1–25.7)	22.2% (19.9–24.3)	54.5% (51.8–57.0)	
40–44	24.2% (21.7–26.6)	23.9% (21.3–26.3)	51.9% (49.2–54.8)	
45–49	24.8% (22.1–27.6)	27.8% (24.9–30.8)	47.4% (44.2–50.4)	
50–54	28.1% (23.9–32.2)	24.5% (20.6–28.7)	47.4% (42.8–52.0)	
55–59	33.2% (27.9–38.9)	25.7% (20.7–30.7)	41.1% (35.4–46.8)	
≥60	30.4% (23.9–37.0)	28.3% (21.7–34.8)	41.3% (34.2–48.9)	
**Urbanity**				<0.0001
Urban	20.5% (19.6–21.3)	22.1% (21.2–23.0)	57.5% (56.4–58.5)	
Rural	22.4% (21.1–23.8)	20.1% (18.8–21.4)	57.5% (55.9–59.1)	

* *p* for overall difference was calculated from Chi–Square tests.

**Table 3:** Changes in the Positive and Negative Affect Schedule (PANAS) in Chinese residents aged 18 years and over during home quarantine induced by COVID-19, 2020.

	PANAS Positive Affect	PANAS Negative Affect
**Total**		
*n* = 12107	24.78 ± 6.88	19.34 ± 7.05
**Sex**		
Male (*n =* 5633)	25.09 ± 7.06	19.04 ± 7.00
Female (*n =* 6474)	24.51 ± 6.70	19.61 ± 7.08
*p* for difference	<0.0001	<0.0001
**Urbanity**		
Urban (*n =* 8407)	24.81 ± 6.85	19.46 ± 7.13
Rural (*n =* 3700)	24.70 ± 6.95	19.08 ± 6.86
*p* for difference	0.420	0.006
**Age**		
<20 (*n =* 854)	26.26 ± 7.61 ^ab^	17.35 ± 6.68 ^ab^
20–24 (*n =* 3896)	24.14 ± 7.17	19.69 ± 7.20
25–29 (*n =* 1461)	24.21 ± 6.86	20.43 ± 7.20
30–34 (*n =* 1428)	24.41 ± 6.66	19.86 ± 7.15 ^b^
35–39 (*n =* 1385)	25.21 ± 6.38 ^ab^	19.93 ± 6.84
40–44 (*n =* 1228)	25.52 ± 6.46 ^ab^	19.21 ± 6.98 ^ab^
45–49 (*n =* 910)	25.05 ± 6.12 ^ab^	18.45 ± 6.42 ^ab^
50–54 (*n =* 481)	25.35 ± 6.56 ^ab^	17.48 ± 6.15 ^ab^
55–59 (*n =* 280)	25.90 ± 6.93 ^ab^	17.32 ± 6.47 ^ab^
≥60 (*n =* 184)	25.97 ± 7.15 ^ab^	17.18 ± 7.46 ^ab^
*p* for difference	<0.0001	<0.0001
**Physical activity level**		
Vigorous (*n =* 2548)	27.54 ± 6.44	18.41 ± 6.49
Moderate (*n =* 2602)	25.53 ± 6.37 *	18.93 ± 6.51 *
Light (*n =* 6957)	23.48 ± 6.88 *^#^	19.34 ± 7.39 *^#^
*p* for difference	<0.0001	<0.0001

PANAS = Positive and Negative Affect Schedule. ^a^ *p* < 0.05 versus 20–24; ^b^ *p* < 0.05 versus 25–29; * *p* < 0.05 versus Vigorous level; ^#^ *p* < 0.05 versus Moderate level. All values were presented as mean ± SD.

## Appendix

**Table A1:** Targeted survey sampling number per each province among Chinese residents aged 18 years and over during home quarantine induced by COVID-19 in China, 2020.

Province	Targeted Survey Sampling Number	Total Population Size
Guangdong	600 ± 200	Large sample
Shandong	600 ± 200	Large sample
Henan	600 ± 200	Large sample
Sichuan	600 ± 200	Large sample
Jiangsu	600 ± 200	Large sample
Hebei	600 ± 200	Large sample
Hunan	600 ± 200	Large sample
Anhui	600 ± 200	Large sample
Hubei	600 ± 200	Large sample
Zhejiang	600 ± 200	Large sample
Beijing	600 ± 200	Large sample
Shanghai	600 ± 200	Large sample
Guangxi	350 ± 100	Medium sample
Yunnan	350 ± 100	Medium sample
Jiangxi	350 ± 100	Medium sample
Liaoning	350 ± 100	Medium sample
Heilongjiang	350 ± 100	Medium sample
Shananxi	350 ± 100	Medium sample
Fujian	350 ± 100	Medium sample
Shanxi	350 ± 100	Medium sample
Guizhou	350 ± 100	Medium sample
Chongqing	200 ± 50	Small sample
Jilin	200 ± 50	Small sample
Gansu	200 ± 50	Small sample
Inner mongolia	200 ± 50	Small sample
Xinjiang	200 ± 50	Small sample
Tianjin	200 ± 50	Small sample
Hainan	150 ± 50	Tiny sample
Ningxia	150 ± 50	Tiny sample
Qinghai	150 ± 50	Tiny sample
Tibet	150 ± 50	Tiny sample

**Table A2:** The criteria for these three levels of physical activity.

Category	Criteria
**Vigorous**	meeting at least one of the following criteria (a) vigorous–intensity activity on at least 3 days achieving a minimum of at least 1500 MET–min/week **OR** (b) 7 or more days of any combination of walking, moderate–intensity or vigorous intensity activities achieving a minimum of at least 3000 MET–min/week
**Moderate**	(a) 3 or more days of vigorous activity of at least 25 min per day **OR** (b) 5 or more days of moderate–intensity activity or walking of at least 30 min per day **OR** (c) 5 or more days of any combination of walking, moderate–intensity or vigorous intensity activities achieving a minimum of at least 600 MET–min/week.
**Light**	Those individuals who not meet criteria for Categories 1 or 2

Cited by the guidelines for data processing and analyses of the International Physical Activity Questionnaire (IPAQ) and WHO recommended guideline.

**Table A3:** Provincial levels of insufficient physical activity during home quarantine induced by COVID-19 outbreak in mainland China, 2020.

	Men and Women				Men				Women			
	Total Sample Size	Number of Insufficient Physical Activity	The Prevalence of Insufficient Physical Activity % (95% CI)	Rank	Total Sample Size	Number of Insufficient Physical Activity	The Prevalence of Insufficient Physical Activity % (95% CI)	Rank	Total Sample Size	Number of Insufficient Physical Activity	The Prevalence of Insufficient Physical Activity % (95% CI)	Rank
**Qinghai**	133	98	73.7% (66.2–81.2)	1	60	44	73.3% (61.7–83.3)	4	73	54	74.0% (64.4–83.6)	1
**Xinjiang**	137	95	69.3% (61.3–76.6)	2	48	36	75.0% (60.4–85.4)	2	89	59	66.3% (56.2–75.3)	5
**Jilin**	160	110	68.8% (61.3–75.6)	3	87	66	75.9% (66.7–85.1)	1	73	44	60.3% (49.3–71.2)	17
**Heilongjiang**	220	151	68.6% (62.7–74.5)	4	77	52	67.5% (57.1–77.9)	5	143	99	69.2% (61.5–76.2)	3
**Tibet**	91	61	67.0% (57.1–75.8)	5	39	29	74.4% (59.0–87.2)	3	52	32	61.5% (48.1–73.1)	14
**Guangxi**	330	219	66.4% (61.5–71.2)	6	124	73	58.9% (50.0–67.7)	11	146	206	70.9% (64.1–76.2)	2
**Chongqing**	178	115	64.6% (57.9–71.9)	7	99	64	64.6% (54.5–73.7)	6	79	51	64.6% (54.5–74.7)	8
**Jiangxi**	358	226	63.1% (57.8–68.2)	8	161	97	60.2% (52.8–67.7)	10	197	129	65.5% (58.9–72.1)	7
**Ningxia**	240	151	62.9% (56.3–69.2)	9	119	68	57.1% (47.9–65.5)	14	121	83	68.6% (60.3–76.9)	4
**Liaoning**	314	195	62.1% (56.7–67.2)	10	120	67	55.8% (46.7–64.2)	19	194	128	66.0% (59.3–72.7)	6
**Guizhou**	161	99	61.5% (53.4–68.3)	11	81	49	60.5% (49.4–71.6)	9	80	50	62.5% (51.3–72.5)	12
**Shandong**	952	584	61.3% (58.4–64.4)	12	460	266	57.8% (53.5–62.2)	13	492	318	64.6% (60.6–68.9)	9
**Henan**	685	416	60.7% (57.1–64.5)	13	350	203	58.0% (52.3–62.9)	12	335	213	63.6% (58.5–68.7)	10
**Zhejiang**	860	519	60.3% (57.2–63.7)	14	349	195	55.9% (50.4–60.7)	17	511	324	63.4% (59.3–67.7)	11
**Tianjin**	130	78	60.0% (51.5–68.5)	15	53	30	56.6% (43.4–69.8)	16	77	48	62.3% (51.9–74.0)	13
**Hunan**	544	326	59.9% (55.5–64.0)	16	258	158	61.2% (55.4–67.1)	8	286	168	58.7% (53.1–64.3)	20
**Shanxi**	290	169	58.3% (52.4–64.1)	17	142	79	55.6 (47.2–64.1)	20	148	90	60.8% (52.7–68.9)	15
**Hebei**	561	324	57.8% (53.8–61.5)	18	258	140	54.3 (48.1–60.5)	22	303	184	60.7% (55.1–66.3)	16
**Shananxi**	243	138	56.8% (49.8–63.0)	19	120	68	56.7% (48.3–65.8)	15	123	70	56.9% (48.8–65.9)	21
**Guangdong**	672	374	55.7% (51.9–59.5)	20	314	161	51.3% (45.5–56.7)	24	358	213	59.5% (54.7–64.8)	18
**Gansu**	178	99	55.6% (48.3–62.4)	21	74	38	51.4% (39.2–62.2)	23	104	61	58.7% (49.0–67.3)	19
**Jiangsu**	809	449	55.5% (52.2–58.7)	22	354	198	55.9% (50.8–60.7)	18	455	251	55.2% (50.3–59.3)	23
**Hubei**	361	198	54.8% (49.6–59.8)	23	147	81	55.1% (46.9–62.6)	21	214	117	54.7% (48.1–61.7)	24
**Inner Mongolia**	165	90	54.5% (47.3–61.8)	24	80	49	61.3% (51.3–72.5)	7	85	41	48.2% (37.6–58.8)	31
**Sichuan**	461	238	51.6% (47.1–56.0)	25	219	101	46.1% (39.7–53.4)	30	242	137	56.6% (50.4–62.8)	22
**Yunnan**	264	134	50.8% (45.1–56.4)	26	162	82	50.6% (42.6–58.6)	27	102	52	51.0% (41.2–59.8)	28
**Anhui**	629	316	50.2% (46.3–54.2)	27	337	172	51.0% (45.4–57.0)	25	292	144	49.3% (43.5–55.5)	29
**Shanghai**	957	480	50.2% (47.1–53.5)	28	465	225	48.4% (44.3–53.5)	28	492	255	51.8% (47.6–56.1)	27
**Fujian**	268	134	50.0% (44.0–56.0)	29	123	57	46.3% (37.4–54.5)	29	145	77	53.1% (44.8–60.7)	26
**Hainan**	139	69	49.6% (41.0–57.6)	30	65	33	50.8% (38.5–63.1)	26	74	36	48.6% (36.5–59.5)	30
**Beijing**	617	302	48.9% (45.1–52.7)	31	288	125	43.4% (37.2–49.0)	31	329	177	53.8% (48.3–59.3)	25
**China (total)**	12107	6957	57.5% (56.6–58.3)		5633	3106	55.1% (53.9–56.6)		6474	3851	59.5% (58.2–60.7)	

Rank: The ranking of the prevalence of insufficient physical activity in different provinces.
